# A case report of primary para-testicular spindle cell rhabdomyosarcoma

**DOI:** 10.3389/fonc.2023.1166503

**Published:** 2023-06-07

**Authors:** Peng Su, Ying Yang, Xiaomin Wang, Shulian Chen, Neng Zhang, Hua Yang

**Affiliations:** ^1^ Department of Urology, The Affiliated Hospital of Zunyi Medical University, Zunyi, China; ^2^ Department of Dermatology, The Second Affiliated Hospital of Zunyi Medical University, Zunyi, China; ^3^ Department of Pathology, The Second Affiliated Hospital of Zunyi Medical University, Zunyi, China

**Keywords:** spindle cell rhabdomyosarcoma, para-testicular, pathological features, case report, radical inguinal orchiectomy

## Abstract

Para-testicular rhabdomyosarcoma (PTRMS) is a rare tumor, and it accounts for 7% of all rhabdomyosarcoma tumors. Among all the rhabdomyosarcoma (RMS) types, the spindle cell RMS is extremely rare. The present study describes a case of a para-testicular spindle cell RMS that was treated with a radical inguinal orchiectomy (RIO) and right scrotal resection. A 17-year-old male patient presented with a half-year history of a rapidly growing, painless, right scrotal mass. His CT of the pelvic cavity showed a mixed-density mass in the right scrotum, and the maximum cross-sectional area was approximately 76.5 mm × 64.5 mm. An X-ray of the chest demonstrated no evidence of metastasis, and a local surgical excision was performed subsequently. The histopathological and immunohistochemical examination confirmed the final diagnosis of spindle cell RMS. As a newly diagnosed case, strict and regular follow-up is needed. This article focuses on the importance of prompt recognition, diagnosis, pathological features, and appropriate management of para-testicular spindle cell RMS.

## Introduction

Rhabdomyosarcoma (RMS) is a relatively rare malignant tumor with the most common primary site being the limb. Primary RMS of the genitourinary system accounts for approximately 20% of all cases (1), while para-testicular rhabdomyosarcoma (PTRMS) derived from the interstitial component of the spermatic cord or epididymis is much rarer. According to the clinical characteristics, light microscopic morphology, and cellular and molecular genetics of the tumor, RMS is divided into four categories in the 2013 WHO Classification of Soft Tissue and Bone Tumors: embryonal rhabdomyosarcoma (ERMS), alveolar rhabdomyosarcoma (ARMS), pleomorphic rhabdomyosarcoma (PRMS), and spindle cell/sclerosing rhabdomyosarcoma (SSRMS). The most common histological subtype of RMS is ERMS, while spindle cell RMS accounts for only approximately 5% (2). Spindle cell RMS most occurs in young men, often involving the para-testicular region. Para-testicular spindle cell RMS generally presents as a painless mass that rapidly enlarges in a short period of time, and the tumor is most commonly found in the testis and peri-testicular tissue. The mass can be located in the spermatic cord and epididymis and may be accompanied by hydrocele. The early mass is clearly demarcated from the testis and is often misdiagnosed as a benign lesion. However, in the advanced stage, the mass can invade the testis, and once metastasis occurs, most patients manifest as inguinal lymphadenopathy. Because of the superficial location of the tumor, early surgery is relatively easy and can significantly improve its prognosis. Delayed surgery leads to the deterioration of the disease and increases its mortality rate (3). Therefore, timely diagnosis and treatment are very important.

## Case presentation

A 17-year-old unmarried male patient with no past medical or family cancer history presented to our hospital with a rapidly increasing but painless mass in his right hemi-scrotum. He denied any difficulty in urination, dysuria, or hematuria. He also denied any history of recent trauma, sexually transmitted diseases, or erectile dysfunction. Physical examination confirmed that the mass was approximately 6.0 cm × 6.0 cm in size, felt firm, and had a negative transillumination test. No abnormalities were found in the left scrotum and left testis.

**Figure 1:**
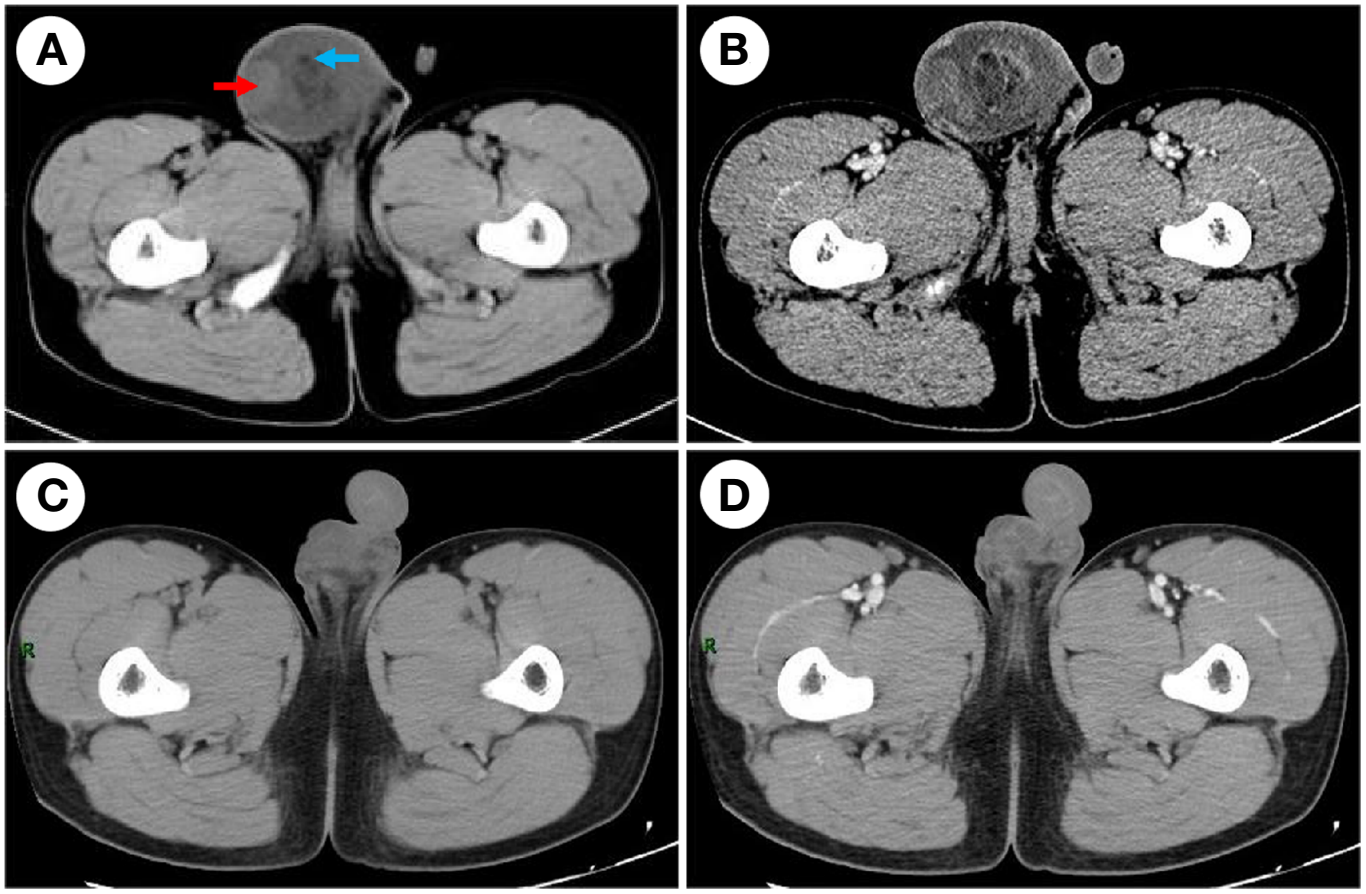
CT examination. **(A)** The CT scan before the surgery shows a mixed-density mass in the right scrotum, watery density (red arrow), and fat density (blue arrow) in the mass. **(B)** Enhanced CT before the surgery shows uneven reinforcement. **(C)** The CT scan after the surgery. **(D)** Enhanced CT after the surgery; the mass was completely removed.

A 52 mm × 60 mm slightly hyperechoic mass in the right scrotum and hydrocele in the right testis were observed by scrotal ultrasound. Pelvic CT revealed a mixed-density mass in the right scrotum with a maximum cross-sectional area of approximately 76.5 mm × 64.5 mm and watery density and fat density in the mass, and enhanced scanning showed uneven reinforcement (**Figures 1A, B**). Several tumor marker levels, including serum alpha-fetoprotein (AFP), human chorionic gonadotrophin (β-HCG), carcinoembryonic antigen (CEA), cortisol (COR), carbohydrate antigen 19-9 (CA19-9), carbohydrate antigen 125 (CA125), and carbohydrate antigen 15-3 (CA15-3), were all in the normal ranges. An X-ray of the chest demonstrated no evidence of metastasis.

**Figure 2:**
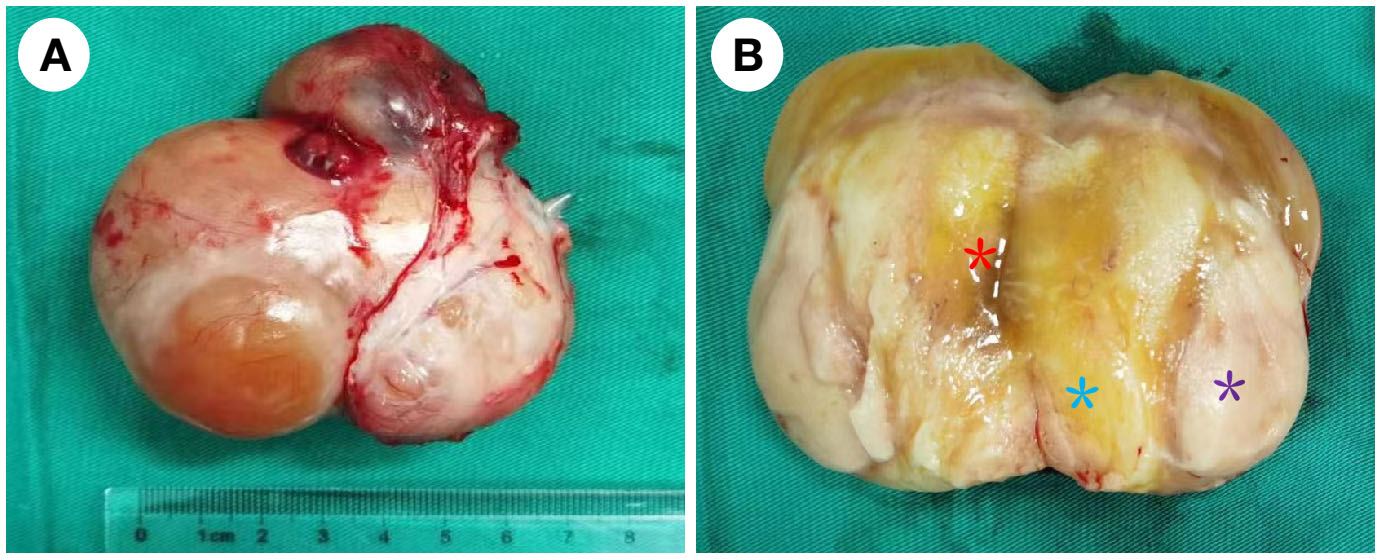
Para-testicular mass after first excision. **(A)** The smooth appearance of the para-testicular SRMS; the size was approximately 6 cm × 7 cm × 4 cm, and its envelope is intact. **(B)** The cut surface of the tumor. It mainly consisted of two parts: grayish-yellow tissue and fish-flesh appearance tissue. The central part of the tumor was mainly grayish-yellow tissue (blue *) with mild liquefaction (red *) visible, and the surrounding part was mainly grayish-white, fine-textured fish-flesh appearance tissue (purple *), and the two parts are growing in a fused pattern with no clear demarcation.

**Figure 3:**
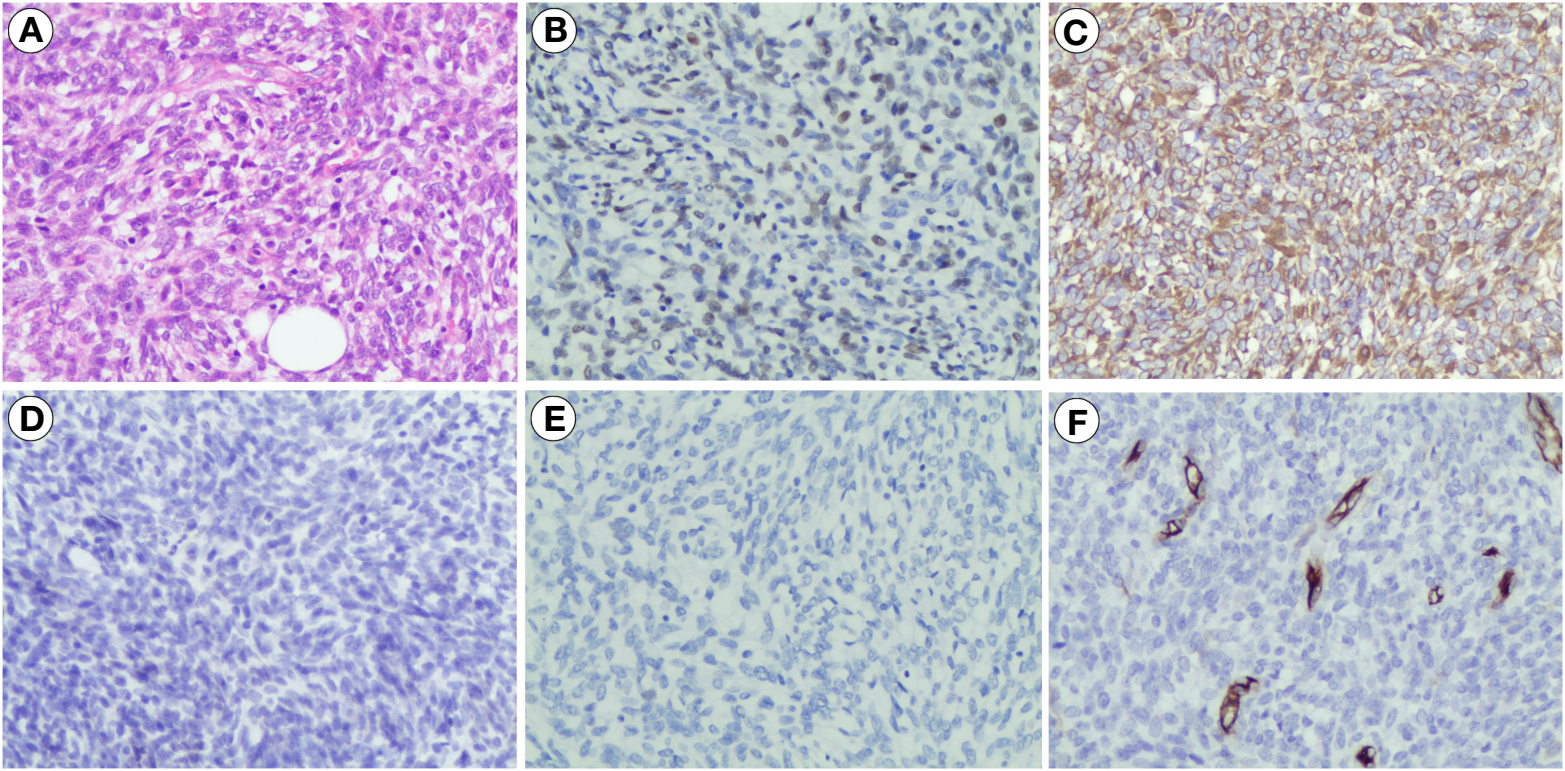
Pathological features of para-testicular SRMS. **(A)** Histopathological section (hematoxylin and eosin staining), ×200. **(B)** Immunohistochemistry results showing the expression of MyoD1 (+), ×200. **(C)** Immunohistochemistry results showing the expression of desmin (+), ×200. **(D)** Immunohistochemistry results showing the expression of S-100 (−), ×200. **(E)** Immunohistochemistry results showing the expression of myogenin (−), ×200. **(F)** Immunohistochemistry results showing the expression of CD34 (−), ×200. SSRMS, spindle cell/sclerosing rhabdomyosarcoma.

Because the mass is clearly demarcated from the testicle and epididymis, local surgical excision was performed, and the mass was completely removed during the first operation, measuring approximately 6 cm × 7 cm × 4 cm. The macroscopic appearance of the tumor section has distinct heterogenicity. The tumor mainly consisted of two parts, grayish-yellow tissue and fish-flesh tissue, with indistinct internal demarcation and fusion with each other. The central part of the tumor was mainly grayish-yellow tissue with mild liquefaction visible, and the surrounding part was mainly grayish-white, fine-textured fish-flesh appearance tissue (**Figures 2A, B**). Postoperative pathological reports from our hospital considered the result to be a possible spindle cell lipoma or spindle cell liposarcoma. Then, the pathological section was sent to Southwest Hospital for pathological consultation, and the report suggested a large number of spindle cells proliferated within the tumor, with moderately atypical, diffusely, or interlaced cells, intercellular collagen fiber proliferation, and some spindle cells infiltrating into adipose tissue. The mitotic count was two nuclear divisions per 10 high-power field (HPF), and no obvious necrosis was found. A large area of hemorrhage was visible in the tumor area, some areas were relatively loose in structure, and there was no excessive mitosis or necrosis in the tumor tissue, indicating a low malignancy. Immunohistochemically, the results were as follows: myogenin (−), MyoD1 (+), desmin (+), CD34 (−), S-100 (−), Ki-67 (30%+), CD99 (−), MDM2 (+), SMA (Vessel+), and BCoR (−) (**Figures 3A–F**), but the fluorescence *in situ* hybridization (FISH) test of *MDM2* gene is negative. The results suggest that the diagnosis was “para-testicular spindle cell RMS” whose prognosis is relatively well. One month later, abdominal lymph node color ultrasound and abdominal CT of the patient showed that the bilateral inguinal and pelvic lymph nodes had no metastasis or recurrence (**Figures 1C, D**). For better treatment, right radical inguinal orchiectomy (RIO) with right scrotal resection was further performed. Postoperative pathology revealed no tumor tissue, and the margins of resection were negative for malignancy.

After the surgery, we recommended that the patient be administered pediatric VAC (vincristine, actinomycin D, and cyclophosphamide) regimen and that sperm cryopreservation be performed before chemoradiotherapy, but the patient refused due to personal reasons. The last follow-up was 9 months after the second operation, and the patient’s abdominal CT did not reveal any local recurrence or distant metastases.

## Discussion

RMS is one of the most common pediatric soft tissue sarcomas, accounting for approximately 40%–60% of all pediatric soft tissue sarcomas (4). Spindle cell RMS was first introduced by the German–Italian Cooperative Sarcoma Study to distinguish it from the ERMS based on its distinctive clinicopathological features and better outcome (5), and it is mainly located in the para-testicular region characterized by a fascicular proliferation of spindle cells. Spindle cell RMS was described as one of the para-testicular tumors that have an excellent outcome and a relatively good prognosis.

### Diagnosis

Para-testicular spindle cell RMS is more common in children and adolescents with non-specific clinical manifestations that present as a rapidly growing, painless, soft tissue mass generally and rarely invading the scrotal skin. Because of its superficial location, it can be completely surgically removed if diagnosed early. However, delaying diagnosis leads to a significant worsening of the prognosis and increases mortality, so self-examination is necessary for adolescents.

In terms of auxiliary inspection, ultrasonography is the most commonly used imaging diagnostic method. The lesion is typically hypoechoic and may demonstrate increased vascularity on Doppler ultrasonography. The mass may be associated with a hydrocele, which may lead to a diagnosis of epididymitis rather than malignancy (6). In this instance, MRI is more useful, as it provides a better characterization of tissue and can be used to differentiate benign and metastatic lesions with high accuracy. The MRI of para-testicular spindle cell RMS shows the following signals: T1-weighted (T1W), isointense to the testicle; T2-weighted (T2W), heterogeneously hyperintense to the testicle; T1W fat-suppressed + contrast-enhanced (FS+CE), heterogeneous enhancement; diffusion-weighted imaging (DWI) diffusion, no restriction owing to necrosis or myxoid component; dynamic contrast-enhanced (DCE) curve profiles, type III or IV (7). However, none of the above tests can confirm the diagnosis, as they only are the main methods to examine the localization of the lesion. Pathology and immunohistochemistry are accurate diagnostic methods.

For this patient, we did a local excision surgery for the first time and did not perform an intraoperative frozen section because the mass was not very large, and the envelope was intact with clear anatomy of the surrounding tissues, allowing for a complete excision. Intraoperative frozen sections should be taken into consideration if a testicular preservation surgery is desired; however, it has limitations and does not always provide an accurate diagnosis.

### Histopathological characteristics

Gross dissection of spindle cell RMS often shows a white or tan whorled appearance, accompanied by necrosis or cystic degeneration occasionally (8). The histopathologic aspects of spindle cell RMS often show fascicular elongated cells with central nuclei and eosinophilic fibrillary cytoplasm, with a small proportion of rhabdomyoblasts, characterized by more eccentric nuclei, striations, and sharper eosinophilia. Interspersed collagen fibers have also been noted. Immature rhabdomyoblasts are admixed among these spindle cells (9). Immunohistochemistry also plays a pivotal role in the identification of this lesion. Generally, spindle cell RMS reacts with myogenic markers, such as desmin and MyoD1. The proportion of desmin-positive cells may vary from case to case depending on the number of differentiated cells, and desmin expression is usually mirrored by muscle-specific actin immunoreactivity (8). However, not all spindle cell RMS tumors are positive for both of the abovementioned immunohistochemical abnormalities. For example, some adult spindle cell RMS presented myogenin (−) and desmin (+) (10). In this case, the patient showed myogenin (−), MyoD1 (+), and desmin (+) as an atypical presentation. In addition, the negative reactivity of S-100 and CD34 helps in the differential diagnosis of other types of sarcomas (8). Therefore, the correct diagnosis must be obtained by pathomorphology combined with immunohistochemistry and clinical features.

Molecular genetic studies of SRMS have made rapid progress in recent years. Indeed, MYOD1 mutations are the most common genetic abnormality in pediatric spindle cell RMS, with an incidence of 67% in children older than 1 year, and can be used as a molecular diagnostic test to stratify these high-risk patients (11). Despite many treatment modalities, MYOD1-mutated tumors tend to be highly aggressive and have a high mortality rate. We did not perform the FISH test and next-generation sequencing (NGS) for MOYD1 molecular detection this time because the patient was older.

### Differential diagnosis

The clinical presentation of RMS is mostly non-specific, and therefore, caution should be used in managing rapidly enlarging painless masses in clinical practice, and the possibility of this disease should be considered. Spindle cell RMS should be differentiated from the following disease. 1) ERMS: ERMS often presents as a mass occurring in the genitourinary tract, head and neck, or abdomen of infants and children younger than 5 years. The 5-year survival rate of the conventional type is 66%. Histologically, the ERMS manifests as the existence of poorly differentiated cells and rhabdomyoblasts, with abundant eosinophilic cytoplasm indicating embryonal rhabdomyosarcoma. By immunohistochemistry, the tumor cells are variably positive with myogenin and desmin. 2) Infantile fibromatosis: it occurs in children less than 2 years old, and the tumors mostly originate from skeletal muscles. Microscopically, they present as fibroblasts in different stages of differentiation and mainly round, oval, or short spindle cells arranged in sheets or bundles. Immunohistochemically, the tumor cells can express SMA in varying degrees, but not myogenic markers. 3) Para-testicular liposarcoma (PLS): it mostly originates from the spermatic cord, testicular membrane, or epididymis; usually presents as a painless inguinal or scrotal soft tissue mass; and is often misdiagnosed as an inguinal hernia or testicular tumor. Patients with PLS are usually older than those with SRMS. Histologically, PLS is generally considered to stain positive for MDM2 and S-100, and the FISH exam shows multiple copies of *MDM2* gene with clustered amplification. The patient we report was not excluded from the diagnosis of liposarcoma at the first time due to positive MDM2 immunohistochemistry, but later, his FISH test for *MDM2* gene was negative. In addition, the patient presented with negative S-100, which is also inconsistent with liposarcoma, in which S-100 can be detected in adipose differentiated cells. Ultimately, we ruled out the diagnosis of liposarcoma.

### Prognosis and treatment

The most significant feature of para-testicular spindle cell RMS is the better prognosis. The prognosis of pediatric SRMS is good, and tumors in para-testicular appear to have a better prognosis than tumors in non-para-testicular sites (8), which may be related to this location being easier to find. Although this patient did not receive chemotherapy, he remained healthy at the 9-month post-operative follow-up, and the patient was satisfied with the treatment outcome. We want to point out that the major factors affecting the prognosis of SRMS are resectability, tumor size (which is also associated with resectability), histologic subtype, and tumor stage (12).

In terms of treatment, owing to its rarity, spindle cell RMS is treated aggressively with similar protocols as other RMS types. The standard protocols involve combined therapy including radical inguinal orchiectomy, chemotherapy, and adjuvant radiation (13, 14). Moreover, reproductive endocrine function is also very important (15). In this case, due to an insufficient understanding of this disease, only scrotal mass resection was performed during the initial surgery. Postoperative pathological results considered the possibility of spindle cell lipoma or spindle cell liposarcoma, and no further treatment was given. After the diagnosis of spindle cell RMS was confirmed by pathological consultation outside, abdominal CT and inguinal color Doppler ultrasonography showed no bilateral inguinal and pelvic lymph node metastasis, and then the “radical inguinal orchiectomy of the right testis and epididymis through the groin + right scrotal resection” was further performed. Postoperative systemic chemotherapy is required to further reduce the recurrence rate and improve the survival rate. For example, the VAC regimen and IVA (ifosfamide + vincristine + adriamycin) regimen are commonly used (16). With the continuous discovery of tumorigenic genes, studies found that many targeted drugs may be helpful for SRMS treatment, but most drugs are still under study, with few having entered clinical trials (14). Biological immunotherapy, as one of the emerging regimens for cancer therapy, can also regulate the immune tolerance status of patients on the basis of assisting in killing tumor cells.

## Conclusion

Para-testicular spindle cell RMS is a rare tumor occurring in children and adolescents. Histopathological characteristics are similar to those of other spindle cell neoplasms, so the correct diagnosis mainly relies on clinical manifestations and histopathological characteristics. In this article, we have described the diagnosis, pathology, treatments, and prognosis of the spindle cell RMS to raise people’s awareness of this rare disease in order to improve the accuracy of diagnosis and reduce the pain of patients.

## Data availability statement

The original contributions presented in the study are included in the article/Supplementary Material. Further inquiries can be directed to the corresponding authors.

## Ethics statement

The studies involving human participants were reviewed and approved by The Ethics Committee of the Affiliated Hospital of Zunyi Medical University. Written informed consent was obtained from the participant/patient(s) for the publication of this case report.

## Author contributions

PS was the patient’s urologist. HY was the patient’s pathologist. XW and YY contributed to the manuscript drafting. SC and NZ were responsible for the revision of the manuscript and important intellectual content. All authors contributed to the article and approved the submitted version.
